# Comparison of Saudi Pharmacist Licensure Examination (SPLE) Pass Rates by Institution and Applicant Characteristics

**DOI:** 10.3390/healthcare10101865

**Published:** 2022-09-24

**Authors:** Wael A. Alghamdi, Tahani M. Almeleebia, Khalid M. Orayj

**Affiliations:** Department of Clinical Pharmacy, College of Pharmacy, King Khalid University, Abha 61441, Saudi Arabia

**Keywords:** pass rate, pharmacy education, pharmacy licensure, SPLE

## Abstract

In 2019, the Saudi Pharmacist Licensure Examination (SPLE) was first administered to all pharmacy graduates and served as one of the prerequisites for obtaining a pharmacist license. The objective of this study was to evaluate whether institution and applicant characteristics are associated with first-time SPLE success. Passing status for 2284 SPLE first-time applicants was obtained from online public data for the years 2019 and 2020. The data included applicant sex, institution type (public vs. private), and college establishment year (2006 or earlier vs. after 2006). Overall, the SPLE first-time pass rate in 2020 was significantly higher than in 2019 (98.0 vs. 95.9%; *p* = 0.0062). Applicants from pharmacy colleges established in or before 2006 had a higher SPLE first-time pass rate, compared to those from pharmacy colleges established after 2006 (98.2 vs. 95.2%; *p* < 0.0001). The pass rate for male applicants was lower compared to female applicants (95.8 vs. 97.5%; *p* = 0.0221). The results of logistic regression showed that exam year (2020 vs. 2019), applicant sex (female vs. male), and pharmacy college establishment year (≤2006 vs. >2006) were statistically significant predictors. Further studies are needed in the upcoming years when more cumulative data are available.

## 1. Introduction

The growing population of Saudi Arabia and the increasing number of healthcare institutions are boosting the demand for pharmaceutical services. The demand for pharmacists in the job market led to establishing new colleges of pharmacy across Saudi Arabia [1]. The number of pharmacy colleges has expanded rapidly, from 10 in 2005, to 22 in 2008, and 30 in 2014. As a result, the number of pharmacy graduates has increased from 246 graduates in 2006 to 768 graduates in 2014 and more than 1900 graduates in 2019 [2]. The rapid and dramatic increase in pharmacy graduates emphasized the need to set standards for pharmacist licensure to ensure the safety of the public.

The Saudi Commission for Health Specialties (SCFHS) was established in 1992 to set standards for healthcare-related practices and accreditation for postgraduate training programs. In 2019, the SCFHS began administering the Saudi Pharmacist Licensure Examination (SPLE) to all pharmacy graduates and served as one of the prerequisites to obtaining a license and practicing pharmacy in Saudi Arabia. The goal of the licensure exam is to ensure that pharmacists have the required competencies for safe practice [3]. Additionally, the results of the licensure exam serve as one of the programmatic outcomes that are evaluated internally by the academic institutions, as well as the accreditation bodies.

The United States has a long and rich experience with its pharmacy licensure examination, the North American Pharmacist Licensure Examination (NAPLEX). The first national pharmacy examination was introduced in 1976, and numerous studies have been published to identify predictors for passing the exam [4,5,6,7,8,9]. One of the studies that examined the impact of pharmacy school characteristics on the NAPLEX first-time pass rate was by Jimenez et al. They reported that a public institution and a graduating class size of 100 or more were significant predictors of NAPLEX high pass rates [5]. Additionally, Williams et al. reported that a higher NAPLEX first-time pass rate was correlated with public institutions and colleges located within an academic health center [6]. Other studies explored the association between student characteristics and NAPLEX first-time pass rate, such as the one by McCall et al., who reported that a higher grade point average (GPA), younger age, and higher Pharmacy College Admissions Test (PCAT) scores were associated with higher success rates [7]. Other variables, such as race/ethnicity, pharmacy GPA, and on-time graduation, were also reported as predictors of passing NAPLEX [8].

In Saudi Arabia, the scarcity of research in this field is most likely due to the new experience with SPLE. Alhifany et al. concluded from a single-institution cross-sectional study that a high SPLE score was significantly associated with applicant sex, the GPA of pharmacology courses, and the GPA of therapeutics courses [10]. A large-scale study comprising graduates from multiple colleges of pharmacy across the Kingdom of Saudi Arabia is needed. The objective of this study was to evaluate whether institution and applicant characteristics, including exam year, college type, college establishment year, and applicant sex, are associated with first-time SPLE pass rate.

## 2. Methods

### 2.1. Data Collection

This is a cross-sectional study, in which first-time SPLE pass rates were obtained from online public data available on the Saudi Commission for Health Specialties website [11]. The data were collected for the years 2019 and 2020 since the SPLE was launched in 2019; by the time of the study, the data for 2021 were not complete. While obtaining data from the public SCFHS dashboard, the ‘attempts’ filter was used to obtain first-time applicants and the ‘exam year’ filter was used to limit the results to applicants from 2019 and 2020. Other filters, such as ‘university type’, ‘university’, and ‘sex’, were also used to obtain additional data on the applicants. The data included applicants from all colleges regardless of the number of applicants per college. The collected data included applicant sex and institution type (public vs. private). Additional data on pharmacy colleges were obtained, such as the college region and its established year (2006 or earlier vs. after 2006). The number of new pharmacy colleges increased rapidly after 2006 [12], hence why this was included in the analysis as a potential variable.

### 2.2. Statistical Analysis

Categorical variables were summarized as frequencies and percentages. The Chi-squared test was used to examine the association of collected categorical variables with SPLE first-time pass status (i.e., passed or failed). A stepwise regression using a backward procedure was performed to develop multiple logistic models. In the stepwise approach, the *p*-value was set to 0.05 as the exclusion criteria for individual variables. Statistical analyses were performed using JMP^®^ Pro v16 (SAS Institute Inc., Cary, NC, USA).

## 3. Results

The passing status for 2284 SPLE first-time applicants was obtained. The applicants were from 27 pharmacy colleges and sat for the SPLE exam during 2019 and 2020 (Table S1). A larger number of applicants sat for the SPLE exam during 2019 (*n* = 1302) compared to 2020 (*n* = 982) (Table 1). About 88% of the applicants were from public institutions, and 61% were female. The number of applicants from colleges established in 2006 or earlier was 1248 (55%), compared to 1036 (45%) applicants from colleges established after 2006. Over 50% of the applicants graduated from colleges located in the Riyadh and Mecca regions.

Overall, the SPLE first-time pass rate in 2020 was significantly higher than in 2019 (98.0 vs. 95.9%; *p* = 0.0062) (Table 2). During 2019 and 2020, applicants from pharmacy colleges that were established in 2006 or earlier had a higher SPLE first-time pass rate, compared to applicants from pharmacy colleges established after 2006 (98.2 vs. 95.2%; *p* < 0.0001). The pass rate for male applicants was lower compared to female applicants (95.8 vs. 97.5%; *p* = 0.0221). Additionally, the pass rate for applicants from public colleges was slightly higher than private colleges with a borderline significance (*p* = 0.062). In 2019 alone, applicants from pharmacy colleges that were established in 2006 or earlier had a 97.7% pass rate, while it was 93.8% (*p* = 0.0004) for applicants from colleges established after 2006. In 2020, the pass rate for female applicants was higher than for male applicants (99.1 vs. 96.5%; *p* = 0.0037).

When a stepwise logistic regression was performed for the combined 2019 and 2020 data, all three variables (exam year, applicant sex, and COP establishment year) were statistically significant, hence they were included in the model (Table 3). The odds ratio for first-time applicants in 2020, compared to applicants in 2019, was 2.2 (95% CI, 1.3–3.6; *p* = 0.0043). In addition, male applicants had lower odds of passing the exam (OR 0.50; 95% CI, 0.31–0.80; *p* = 0.0042), while applicants from COPs established in 2006 or earlier had higher odds of passing the exam (OR 2.9; 95% CI, 1.7–4.8; *p* < 0.0001).

## 4. Discussion

To our knowledge, this is the first study to analyze SPLE pass rates using public data provided by SCFHS. During 2019 and 2020, the overall SPLE first-time pass rate exceeded 95%. Our analysis showed several characteristics that were associated with SPLE first-time pass rates, including the pharmacy college establishment year (i.e., 2006 or earlier vs. after 2006), institution type, and applicant sex.

Pharmacy college establishment year was shown as a significant factor when the analysis included 2019 data and the combined 2019 and 2020 data. A possible explanation is that older universities may have cumulative teaching experience, more learning resources, and financial support that might have contributed to providing high-quality education. A similar trend has been found in the US where applicants from older pharmacy colleges had higher pass rates. Williams et al. found that applicants from U.S. pharmacy colleges established before 2000 had higher pass rates compared to those from colleges established in 2000 or after [6]. They noted that those colleges established before 2000 tended to be public, located at academic health centers, and had the traditional four-year program structure, hence the reason for their difference may have come from one or more of those four characteristics. Lebovitz et al. also reported a similar trend with US pharmacy colleges established before and after 1995 [13]. They also noted that a higher percentage of the older colleges were public colleges.

In our analysis of the SPLE first-time pass rate, there was a borderline significant association between the first-time pass rate and graduating from a public versus private college (*p* = 0.062). A similar trend has been reported in the US as well [5,6,9,13]. In Saudi Arabia, public education (including universities) is free for all Saudi nationals [14], hence public universities are considered a preferred choice for the majority of high school graduates compared to private schools; this, along with the already low number of private schools, may explain the high number of applicants from public universities, compared to applicants from private schools. In addition, public universities are well funded by the government. In 2019, the Saudi government spent about USD51 billion in the education sector (including public universities), accounting for 18% of government spending, which could explain, in part, the observed trend [15].

Our logistic regression results showed that male applicants were less likely to pass the SPLE compared to females, although the pass rate difference was not large (95.8 vs. 97.5%, respectively). In a review of the predictors of success for NAPLEX by Park et al., they included six studies in their review that looked at applicant sex as a predictor [9]; none of the studies found a difference except for one study that reported that the mean NAPLEX score was higher by 1.65 points (*p* = 0.017) in males compared to females. Due to the relatively short age of the SPLE test and the nature of the many colleges where males and females are being taught somewhat separately, this requires further studies to examine the effect of sex in greater depth.

Of interest, the year in which the SPLE exam was conducted was also a predictor of the first-time pass rate. The pass rate was higher in 2020 compared to 2019. A possible explanation is that individuals who sat for SPLE in 2019 may have shared the exam questions with the upcoming applicants. Questions that were said to have appeared on the SPLE are circulating among students on social networks such as WhatsApp and Telegram.

It is worth comparing SPLE first-time pass rates (95.5% in 2019 and 97.7% in 2020) with the NAPLEX first-time pass rates, which were 88.3% in 2019 and 88.4% in 2020 [16]. Clearly, the two exams cannot be compared in terms of their difficulty, since they are different in many aspects, especially in their competency statements [3,17]. However, comparing pass rates and exam competency statements with licensure examinations from other countries might be useful, given the recent implementation of SPLE and the lack of historical data to ensure whether the exam goal is being achieved.

A limitation of this study is the lack of additional variables that might have affected the results; however, every possible public resource was utilized to extract information related to applicant universities. Individualized data (e.g., age, GPA, graduating on time, etc.) were not possible to collect from all universities. Other limitations include other factors that are beyond the scope of this study that could have influenced the results and the data collection for two years only, given the recent implementation of SPLE.

## 5. Conclusions

To summarize, the study has shed light on the passing rates of applicants for the SPLE test and indicated some of the possible factors that affected passing rates, including exam year, pharmacy college establishment year (≤2006 vs. >2006), and applicant sex. SPLE has just been implemented recently, so further studies are needed in the upcoming years when more cumulative data are available.

## Figures and Tables

**Table 1:** Characteristics of first-time SPLE applicants during 2019 and 2020.

Characteristic	Number of Applicants, *n* (%) (*n* = 2284)
Year	
2019	1302 (57.0)
2020	982 (43.0)
Sex	
Male	895 (39.2)
Female	1389 (60.8)
COP establishment year	
2006 or earlier	1248 (54.6)
after 2006	1036 (45.4)
Institution type	
Public	2006 (87.8)
Private	278 (12.2)
University region	
Riyadh	756 (33.1)
Mecca	494 (21.6)
Eastern	271 (11.9)
Qassim	215 (9.4)
Jizan	171 (7.5)
Asir	167 (7.3)
Medina	71 (3.1)
Northern Borders	49 (2.2)
Albaha	36 (1.6)
Tabuk	21 (0.9)
Hail	17 (0.7)
Jouf	9 (0.4)
Najran	7 (0.3)

COP: college of pharmacy.

**Table 2:** SPLE first-time pass rates per applicant and institution characteristics.

Characteristic	2019		2020		Combined (2019 and 2020)	
	Pass Rate	*p*-Value *	Pass Rate	*p*-Value *	Pass Rate	*p*-Value *
Year						
2019	NA		NA		95.9	0.0062
2020					98.0	
Sex						
Male	95.1	0.2640	96.5	0.0037	95.8	0.0221
Female	96.4		99.1		97.5	
Institution type						
Public	96.3	0.0590	98.2	0.2470	97.1	0.0628
Private	92.7		96.8		95.0	
COP establishment year						
2006 or before	97.7	0.0004	98.7	0.0610	98.2	<0.0001
after 2006	93.8		97.0		95.2	

* Chi-squared test. COP: college of pharmacy.

**Table 3:** Multiple logistic regression models.

Characteristic	2019		2020		Combined (2019, 2020)	
	OR (95% CI)	*p*-Value	OR (95% CI)	*p*-Value	OR (95% CI)	*p*-Value
Year, 2020	NA		NA		2.2 (1.3–3.6)	0.0043
Sex, male	-		0.25 (0.09–0.68)	0.0071	0.50 (0.31–0.80)	0.0042
COP establishment, >2006	2.8 (1.6–5.1)	0.0007	-		2.9 (1.7–4.8)	<0.0001

COP: college of pharmacy.

## Data Availability

No new data were created in this study. Data sharing is not applicable to this article.

## Supplementary Materials

The following are available online at https://www.mdpi.com/article/10.3390/healthcare10101865/s1, Table S1: SPLE pass rates extracted from SCFHS website.
