# Supplementary material for: Comparison of Saudi Pharmacist Licensure Examination (SPLE) Pass Rates by Institution and Applicant Characteristics
Source: Healthcare (Basel). 2022 Sep 24;10(10):1865. doi: 10.3390/healthcare10101865 (PMC9602314; doi:10.3390/healthcare10101865)
Supplement: Supplementary file 1 [file healthcare-10-01865-s001.zip › healthcare-1926993-supplementary.pdf]

SUPPLEMENTARY MATERIALS

Table S1. SPLE pass rates extracted from SCFHS website (accessed on December 8, 2021).

**University**	**2019**				**2020**			
	first-time attempts	pass rate, %	all attempt s	pass rate, %	first-time attempts	pass rate, %	all attempt s	pass rate, %
Albaha University	13	100	19	100	23	100	25	100
Aljouf University	3	100	4	100	6	83	12	92
Almaarefa University	53	92	58	93	33	100	44	93
Batterjee Medical College	3	100	4	100	12	100	15	100
Buraydah Colleges	11	82	12	83	7	100	14	86
Ibn Sina College for Medical Studies	27	100	39	97	79	97	85	96
Imam Abdulrahman bin Faisal University	76	100	82	100	73	100	79	100
Jazan University	87	92	117	91	84	95	102	95
King Abdulaziz University	80	99	88	99	72	100	81	100
King Faisal University	68	100	70	100	32	100	37	97
King Khalid University	87	92	116	93	80	98	116	97
King Saud Bin Abdulaziz University for Health Sciences	70	100	77	100	33	100	34	100
King Saud University	96	100	113	100	92	99	114	99
Mohammad Al-Mana College for	12	92	18	94	10	80	11	82

Medical Sciences								
Najran University	-	-	-	-	7	100	12	100
Northern Borders University	24	63	34	74	25	92	28	89
Prince Sattam bin Abdulaziz University	15	100	37	100	17	94	29	90
Princess Nora bint Abdulrahman University	195	98	224	97	75	100	102	98
Qassim University - Buraidah	118	100	133	100	35	100	47	98
Qassim University - Unayzah	17	100	22	100	27	100	38	100
Riyadh Elm University	18	89	21	90	13	92	23	96
Shaqra University	23	57	43	70	23	87	49	82
Taibah University	49	98	56	98	22	100	30	97
Taif University	69	96	83	96	49	98	59	97
Umm Al-Qura University	70	97	79	97	33	100	47	98
University of Hail	8	100	13	100	9	100	12	92
University of Tabuk	10	100	13	100	11	100	18	100

There was an applicant with a second attempt (pass rate 100%) from Alfaisal University (Riyadh), and an applicant with a first attempt (pass rate 100%) from Prince Sultan Military College of Health Sciences (Dhahran).
